# Tailoring the Antibody Response to Aggregated Aß Using Novel Alzheimer-Vaccines

**DOI:** 10.1371/journal.pone.0115237

**Published:** 2015-01-22

**Authors:** Markus Mandler, Radmila Santic, Petra Gruber, Yeliz Cinar, Dagmar Pichler, Susanne Aileen Funke, Dieter Willbold, Achim Schneeberger, Walter Schmidt, Frank Mattner

**Affiliations:** 1 AFFiRiS AG, Karl-Farkas-Gasse 22, A-1030, Vienna, Austria; 2 Institute for Structural Biochemistry (Institute of Complex Systems 6), Forschungszentrum Jülich, 52425, Jülich, Germany; Torrey Pines Institute for Molecular Studies, UNITED STATES

## Abstract

Recent evidence suggests Alzheimer-Disease (AD) to be driven by aggregated Aß. Capitalizing on the mechanism of molecular mimicry and applying several selection layers, we screened peptide libraries for moieties inducing antibodies selectively reacting with Aß-aggregates. The technology identified a pool of peptide candidates; two, AFFITOPES AD01 and AD02, were assessed as vaccination antigens and compared to Aβ1-6, the targeted epitope. When conjugated to Keyhole Limpet Hemocyanin (KLH) and adjuvanted with aluminum, all three peptides induced Aß-targeting antibodies (Abs). In contrast to Aß1-6, AD01- or AD02-induced Abs were characterized by selectivity for aggregated forms of Aß and absence of reactivity with related molecules such as Amyloid Precursor Protein (APP)/ secreted APP-alpha (sAPPa). Administration of AFFITOPE-vaccines to APP-transgenic mice was found to reduce their cerebral amyloid burden, the associated neuropathological alterations and to improve their cognitive functions. Thus, the AFFITOME-technology delivers vaccines capable of inducing a distinct Ab response. Their features may be beneficial to AD-patients, a hypothesis currently tested within a phase-II-study.

## Introduction

Alzheimer’s disease (AD) is the most prevalent neurodegenerative disorder currently affecting 28 million people worldwide [1]. It typically presents with a characteristic amnestic dysfunction associated with other cognitive-, behavioral- and neuropsychiatric changes impairing a given individual’s (social) function and ultimately resulting in its death [2]. Available treatments include three acethylcholinesterase inhibitors (AChEI) and one N-Methyl-D-aspartate (NMDA) antagonist. Their effects are small and only symptomatic in nature [3]. Thus, there is a high medical need for a disease-modifying drug.

Accumulation of Amyloid Beta (Aβ) appears to be an early event and central to the disease process. Aβ is a proteolytic fragment of the amyloid precursor protein (APP) [4, 5, 6]. APP-cleavage results in several peptides including Aβ1-40 and Aβ1-42, which are subject to further processing. Recent studies suggest Aβ-variants and aggregates drive the disease process [7, 8].

Immunotherapy offers the possibility to specifically address Aβ-variants and aggregates. However, targeting self-proteins by immunological means bears the risk of autoimmunity [9]. This is exemplified by autoimmune reactions following the administration of cancer vaccines [10]. While regarded as immune privileged, the brain is not excluded from such reactions but represents a relevant target organ as experienced with AN1792 [11] or deduced from the existence of paraneoplastic autoimmune Central Nervous System (CNS) syndromes [12].

With regard to pathological autoimmunity, both cellular- and humoral effector mechanisms need to be considered. Avoidance of T-cell responses against CNS-targets is crucial as demonstrated by AN1792-triggered cases of meningoencephalitis. All second generation AD-vaccines in clinical development, are designed to avoid activation of target-specific T-cells by restricting antigen length to <8 amino acids (aa) or by excluding bona-fide T-cells epitopes (CAD106, ACC001, UB-311, ACI-24 [13, 14, 15]).

The risk of pathological humoral autoimmunity is primarily related to the antigenic epitopes addressed. Efficient control of this risk requires selective targeting of structures exclusively expressed in disease, so called neo-epitopes. The free N-terminus of native, aggregated Aβ is an excellent example of a neo-epitope. Exclusive reactivity to this structure would preclude antibodies (Abs) induced to cross-react with APP and related molecules such as secreted APP-alpha (sAPPa).

Conventional Aß-vaccines [13, 14, 15, 16] are conjugates of an N-terminal Aß-fragment and a carrier. The N-terminus of Aß is accessible in monomers, aggregates and amyloid plaques. Abs elicited by conventional conjugate-vaccines typically fail to discriminate between the various Aß-aggregation states. Given the fact that Aß-monomers possess physiological functions [17, 18, 19, 20] while aggregates exert neurotoxic and synaptotoxic effects [21, 22, 23, 24], a potential benefit of vaccines may require them to elicit Abs selectively addressing Aß-aggregates.

To generate a vaccine that integrates both, targeting the Aß-N-terminus and selective recognition of Aß-aggregates, we devised a technology based on mechanisms of molecular mimicry. Peptide libraries were screened for peptides exhibiting both features. This yielded several hits. Two of them, AD01 and AD02, were characterized in more detail. Both did exhibit the intended specificity, and were found to reduce pathological alterations and to ameliorate behavioral deficits of APP-transgenic Tg2576-mice. Results obtained not only suggested them to be disease-modifying but to have a safety profile superior to conventional Aβ1-6-based vaccines.

## Material and Methods

### AFFITOPE identification and vaccine formulation

AFFITOPE-peptides were identified by screening of peptide libraries (phage display: New England BioLabs, USA; randomized synthetic hexa- and hepta-peptide libraries: Mimotopes Pty., France or MULTIPIN peptide technology), with monoclonal antibodies (mAbs, AFFiRiS, Austria) specific for the free N-terminus of Aß1-40/42. Identified peptides (EMC microcollections, Germany) were conjugated to KLH (Biosyn, Germany) using N-gamma-Maleimidobutyryl-oxysuccinimide ester (GMBS, Thermo Scientific, USA) and adsorbed to Aluminum-hydroxide (ALUM, Brenntag, Denmark). 30µg peptide/vaccine-dose containing 0.1% ALUM were applied to animals.

### Animal experiments

All animal experiments were performed in accordance with the Austrian Animal Experiments Act (TVG2012) using 8–12 week old female C57Bl/6 mice (Charles River, Germany), or Tg2576-mice (Taconic Farms, USA; 129S6/SvEvTac). Experiments were performed under approval numbers: LF1-TVG-22/004-2007; M58/007052/2011/7 and LF1-TVG-22/0102011. General health was checked by modified Smith Kline Beecham, Harwell, Imperial College, Royal London Hospital, phenotype assessment (SHIRPA) primary observational screen [25]. Mice were injected s.c. 3–6 times in monthly or biweekly intervals. Blood was taken in regular intervals, plasma prepared and stored until further use. At study end mice were sacrificed, cerebrospinal fluid (CSF), brains were collected and hemispheres separated. One hemisphere was fixed in 4% Paraformaldehyde (PFA,Sigma Aldrich, USA), dehydrated and paraffin-embedded. Brain tissue was sectioned at 7μM using a sliding microtome (Leitz, Germany) and sections were mounted on Superfrost Plus Slides (Menzel, Germany). The second hemisphere was quick-frozen at -80°C until further extraction.

### Titer determination by ELISA

Standard enzyme-linked immunosorbent assay (ELISA) technology was used to measure levels of vaccine-induced antibodies in plasma and CSF [26]. Substrates used included murine (Anaspec, USA) and human (BACHEM, CH) Aß1-40/42 (each at 5μg/ml), KLH (1μg/ml), recombinant sAPPa (1µg/ml, Sigma-Aldrich, USA), peptide-Bovine serum albumin (BSA) conjugates (1µM), or Aß-aggregates (5µg/ml, immobilized via Streptavidin). Optical density (OD) was measured at 405nm using a micro-well reader (Tecan, CH). ODmax/2 was calculated. For determination of antibody selectivity for different Aß species (monomers,oligomers and fibrils), relative units were calculated as the ratio of OD values for individual measurements: e.g. OD405nm of Oligomer-specific ELISA signals and OD405nm of Monomer-specific ELISA signals. Abs 3A5 (AFFiRiS, Austria), mAbP2-1 (Life-Technologies, USA) and 6E10 (Covance, USA) served as positive controls.

**Preparation and characterisation of Aβ-monomers,-oligomers and—fibrils** Preparation of Aβ-mono and oligomers (<100kd) was performed according to Johansson et al. with slight modifications [27]. Pure C-terminally biotinylated Aß1-42 was used to prepare seedless Aß-monomers. A 1/10 mix of biotinylated and unmodified Aß1-42 (Anaspec, USA) was used for oligomer- and fibril-production. For preparation of Aβ-mono and oligomers Aβ was first resolved in Hexafluoro-2-propanol (HFIP) over night and subsequently removed by vacuum centrifugation. Aß peptides were then resuspended and separated using a Superdex 75-10/300 column (GE Healthcare, UK). Elution of monomers and oligomers was determined by detection at 214nm with oligomers eluting at 8 ml and monomers at 14,5 ml, respectively. Column calibration was done according to manufacturers protocol (LMW Gel Filtration Calibration Kit; GE Healthcare, UK). For fibril preparation Aß peptides were resuspended in 1xPBS and fibrils were assembled by constant rotation of peptide solutions for 24h at 350rpm (37°C). Fibril-preparations were then centrifuged and the pellet was resuspended in elution buffer used for gelfiltration. Aggregation of Aß-species was confirmed by Thioflavin-T, Western- and Dot blot analysis (see Appendix).

### APP-FACS analysis

To test for APP-specific antibodies a Fluorescence-activated cell sorting (FACS) assay based on Chinese hamster ovary (CHO)-cells stably expressing a fusion protein of human APP and enhanced green fluorescent protein (eGFP) (APP-751-EGFP in pCMV-Sport 6, APP: NP_958816, pCMV-Sport 6 eGFP-FLAG-tagged (Gift from J.M.Peters, IMP, Austria)) was used. A mixture of transfected and un-transfected CHO-cells (50% each) was exposed to diluted plasma and analysed for double positive cells (eGFP and APP) with a FACScan (BD Biosciences, USA). mAbP2-1 served as positive control. For each sample 10,000 events were acquired and analysed using CellQuest software (BD Biosciences).

### Behavioral tests

To analyse cognitive dysfunction immunised Tg2576 animals were subjected to Modified Morris water maze task (MWM, with changes) [28] and contextual fear conditioning (CFC, with changes) [29], both analyzed using AnyMaze software (Stoelting Co, USA). MWM was subdivided into cued-, hidden task, and probe-trial. Animals were trained in a tap-water filled 110-cm pool, allowed to swim for 60s with platform occupancy for 10s prior to the next trial. 24h after the hidden training, memory retention was determined in a single 60s probe-trial without a platform. The percent of distance swam and time spent in each quadrant was determined. For CFC, on day 1 mice were placed in the conditioning chamber (AFFiRiS), allowed to habituate for 2 min. and received three 0.8mA foot-shocks in 2 min intervals plus 30s rest. To assess contextual learning on day 2, animals were readmitted to the chamber and monitored for 5 min. with s120-240 chosen as time frame for analysis (time freezing = lack of movement except for respiration). The first two minutes of day 1 were considered as baseline-freezing which was subtracted from day 2 values. Cognitive testing was initiated 4 weeks prior to sacrification with 4 weeks required to complete both cognitive tests for the individual animals including habituation phases at the site of testing.

### Immunohistochemistry (IHC), immunofluorescence (IF) and analysis of cerebral Aß

IHC/IF analysis was done as described previously [26]. Reactivity of vaccine-induced antibodies to Aß and APP was determined using an adapted Tissue Amyloid Plaque Immunoreactivity (TAPIR) analysis [30] on untreated Tg2576- and human AD-brain sections (n = 4, obtained from Novagen, USA (n = 1) or the UCSD ADRC Brain Bank (n = 3); patients analysed were n = 3 female and n = 1 male; females: Braak stage VI and male patient Braak stage V) using plasma samples and an APP-specific mAb (22C11, EMD Millipore, USA) as control. Competition experiments of AD01- and AD02 induced antibodies were performed using specific AFFITOPE-peptides at a final concentration of 10µM. Control antibody used for amyloid staining on human brain sections was the monoclonal antibody BAM10 (Sigma, USA). For murine sections monoclonal antibody 6E10 (Signet, USA) was used as control antibody.

For Aß-specific IF-staining, brain sections of immunized Tg2576 were processed for analysis of amyloid load and incidence of amyloid bearing vessels using mAb 3A5 (AFFiRiS AG, Austria) [26]. All secondary reagents used were obtained from Vector Labs (USA). For TAPIR analysis, color reactions were performed using DAB-substrate Kit. For IF, sections were mounted and counterstained using DAPI-containing VECTASHIELD-HardSet Mounting Medium. Sections were examined using MIRAX-SCAN (Carl Zeiss AG, Germany). AD-like pathology in animals was assessed by determining the total tissue area of coronal cross sections of the total brain as well as the 3A5 positive area on the respective brain sections were determined and the relative cerebral area occupied by amyloid deposits was calculated using a semi-automated area recognition program (eDefiniens Architect XD; www.definiens.com). For analysis three slides/animal and ≤ five individual sections/slide were assessed. Sections carrying tissue artifacts or aberrant staining were excluded. To assess the number of Aß-positive vessels, 3A5 stained sections (n = 3 slides/animal covering cortex and hippocampus and up to five individual sections per slide) have been analysed. Aß-positive vessels were manually counted in sub-regions of the cortex as well as in the hippocampus. Number of positive vessels per mm² was determined.

### Analysis of micro-hemorrhaging

To assess the number of micro-hemorrhages, sections were stained using the Iron Stain Kit (Sigma Aldrich, USA) according to manufacturer’s protocol. 3 slides/animal covering cortex and hippocampus and up to five individual sections per slide have been analysed. Prussian blue-positive spots were manually counted in sub-regions of the cortex as well as in the hippocampus. Number of positive spots per animal was determined.

### Analysis of cerebral levels of Aß by ELISA

The frozen brain hemispheres were thawed and homogenized in homogenisation buffer (50mM HEPES (pH 7,3), 5mM EDTA, with proteinase inhibitor cocktail: Complete Mini, Roche, CH) and centrifugated at 4°C for 30 minutes at 40.000 rpm. The supernatant was aliquoted and stored at -80°C as soluble fraction. The pellet was re-homogenized in Guanidine-HCl buffer (5M Guanidine-HCl, 50mM HEPES (pH 7,3), 5mM EDTA with proteinase inhibitor cocktail: Complete Mini, Roche, CH) and centrifuged at 1600g. The supernatant was dialysed against PBS, aliquoted and stored at -80°C as insoluble fraction. Fractions were analysed for protein content using the Quick Start Bradford Protein Assay according to manufacturer’s protocol (BioRad, USA).

For quantification of Aβ40 and Aß42 peptides in soluble and insoluble fractions, an ELISA analysis was used (Human Amyloid Beta 40 and Human Amyloid Beta 42 ELISA kits, EMD-Milipore, USA), The concentration of amyloid peptides in ng/mg of total protein was calculated for Aß40 and Aß42 in both fractions (soluble and insoluble).

### ELISPOT analysis

Animals (C57Bl/6 mice) were immunized three times in biweekly intervals with AD01-conjugate (30µg net peptide content/mouse/immunization), AD02-conjugate (30µg net peptide content/mouse/immunization) or Ovalbumin (100µg/mouse/immunisation), adjuvanted with CpG/polyR as adjuvant for T-cell stimulation (CpG (ODN1668: 5′ TCC ATG ACG TTC CTG ATG CT 3′, Invivogen, San Diego, USA) 32 μg/mouse; polyR 100 μg/mouse; Sigma-Aldrich). 1 week after the final immunization animals were sacrificed, splenocytes isolated and analysed for the presence of target specific T-cells by ELISPOT analysis. ELISPOT analysis was performed using Ready-SET-Go kits obtained from eBioscience (San Diego, USA) according to the manufacturer´s protocol. Full length Aß1-42 (50μg/ml,), carrier (KLH, 50μg/ml) or short MHC-I or MHC-II restricted Ovalbumin-derived peptides Ova 244 (TEWTSSNVMEERKIKV; MHC class II restricted; 10μg/ml) and Ova 245 (SIINFEKL; MHC class I restricted; 10μg/ml) as positive control for T-cell induction were used for splenocyte restimulation. Stimulated cells were assayed for the secretion of either Interleukin 4 (IL4) or Interferon gamma (IFNg). The stimulation was controlled by application of two positive control stimulators, for IL4 secretion, Phorbol-12-Myristate-13-Acetate (PMA, working conc.: 20nM) and ionomycin (working conc.:750nM) and for IFNg secretion Concanavalin A (ConA); working conc.:10 μg/ml;) were used, respectively.

### Statistical analysis

All experiments were done blind-coded. To determine statistical significance, values were compared using (i) one-way analysis of variance for unpaired samples with Tukey’s Multiple Comparison Tests, (ii) unpaired T-tests with Welch correction or (iii) Kruskal-Wallis-H-Test with Dunn’s Multiple Comparison Tests. For correlation analysis a Spearman-Rank-Correlation has been calculated.

## Results

To generate an Aß-vaccine not activating Aß-specific T-cells but inducing Abs selectively recognizing aggregated Aß and at the same time being specific for the Aß-N-terminus, we screened peptide libraries with Abs applying various selection filters. Specifically, mAbs directed against the N-terminus of intact, full length Aß1-40/42; (aa1-6: DAEFHR) were used to screen 10^9^ peptides from different hexa- and hepta-peptide libraries for 6–7 mer peptides for binding. Specificity of peptide hits was assessed by competition with Aß1-6 (DAEFRH). Several rounds of selection yielded 68 candidates fulfilling both of the above criteria. Comparing the sequence of the n = 68 peptides to the one of native Aß revealed no candidate with only 1aa exchange and a difference of n = 2aa in 16%, n = 3aa in 31%, n = 4aa in 23.5% or n = 5aa in 9%. The remaining 20.5% of the peptides differed at all positions. As a next step, out of the 68 candidates, 17 were randomly picked and tested for their ability to elicit antibodies reacting to the peptides used for immunization (=immunizing peptide) and, at the same time, Aß. To this end, they were coupled to KLH, which served as carrier, adsorbed to aluminum (ALUM), the adjuvant used, and subcutaneously injected into C57BL/6 and Tg2576-mice. While all 17 induced Ig-Abs reactive with the respective peptide, only 14 elicited Abs recognizing Aß1-10 coupled to BSA. Those conjugates were used to mimick binding to Aß-aggregates, as peptide-BSA conjugates should show a local enrichment of Aß-N-termini probably similar to the situation present in full length, native Aß-aggregates. Two examples, AD01 and AD02, characterized by a difference of 50% in their amino acid sequence compared to the targeted Aß epitope, were characterized in more detail and compared to Aß1-6-KLH-vaccine.

### Immunogenicity of AD-AFFITOPEs

**Figure 1:**
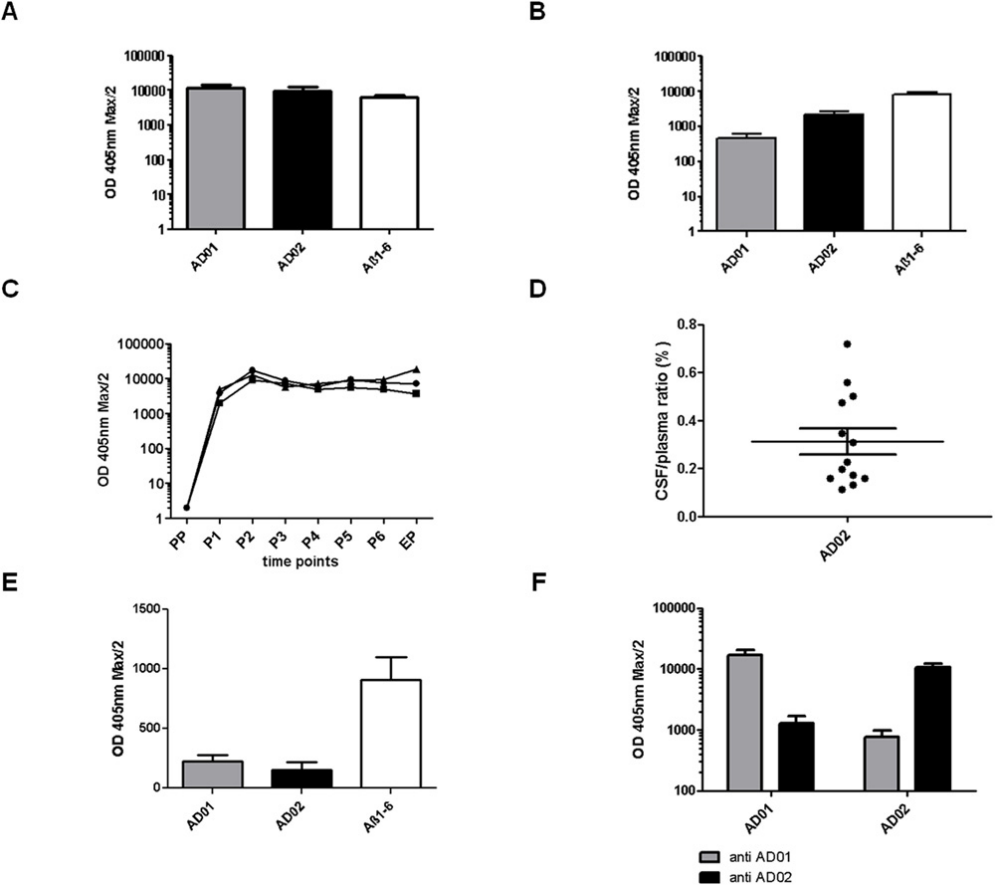
Analysis of the immune response following injection of AD01, AD02 and Aß1-6 conjugate vaccines. Mice were s.c. injected 6 times at a 4-week interval with AD01 (n = 9), AD02 (n = 8) and Aß1-6 conjugate (n = 9) adsorbed to aluminum hydroxide (ALUM). Plasma was taken in monthly intervals and at sacrification. Samples were analyzed for their concentration of IgG Abs against specific peptides. Values depicted are the titer calculated as OD max/2 (at 405nm) plus SEM unless otherwise stated. A) IgG response torwards the respective immunizing peptide (AD01: anti AD01; AD02: anti AD02, Aβ1-6: anti Aβ1-6); B) Reactivity towards human Aβ1-10 after immunization with AD01-, AD02- and Aβ1-6-based conjugate vaccines. Note, none of the 3 vaccines elicits Abs that would react with the Aβ11-19, used as a specificity control (not shown); C) Kinetics of the IgG responses to the immunizing peptide following vaccination with AD01-, AD02- or Aβ1-6 conjugates (AD01… black circle, AD02… black quadrat, Aß1-6… black triangle); D) Ratio of AD02-induced peptide-specific IgG in CSF and plasma. Analysis of AD02-specific IgG levels in CSF and plasma in 13 AD02-immunized animals revealed an average ratio of 0.31% (+/- 0.05%). E) Analysis of sera from vaccinated animals regarding their reactivity towards murine Aβ1-42. Only Aβ1-6 immunized animals show a relevant cross-reactivity to murine Aβ1-42 (Aβ1-6 (n = 9) vs. AD01 (n = 10); p<0.05 and Aβ1-6 (n = 9) vs. AD02 (n = 28); p<0.01); F) IgG response towards the respective immunizing peptide (AD01: anti AD01; AD02: anti AD02) compared to the respective other AFFITOPE peptide (AD01: anti AD02; AD02: anti AD01). Animals included: n = 9 for AD01, n = 8 for AD02.

To test the immunogenicity of AD01 and AD02 in comparison to Aß1-6, Tg2576-mice were injected 6x, s.c., at 4-week intervals with either conjugate-vaccine containing 30µg net peptide. Vaccination induced Abs were measured in plasma samples at defined time points after immunization (AD01 (n = 9), AD02 (n = 8) and Aß1-6 conjugate (n = 9)). All 3 elicited strong and comparable IgG titers towards the peptide used for immunization (Fig 1A). Both AFFITOPEs, AD01 and AD02, elicited Abs to the N-terminus of human Aß at levels comparable to the Aß1-6-KLH conjugate-vaccine (Fig 1B). Of note, the IgG responses triggered by the 3 conjugate-vaccines followed the same kinetics (Fig. 1C). Titers reached a plateau after 2 immunizations, which was stable during the treatment period. Analyzing the CSF of AD02-immunized Tg2576-mice demonstrated the presence of peptide-/Aß-specific Abs at a level of 0.1–0.7% (0.31% +/- 0.05%) of the respective plasma levels (Fig. 1D).

### Specificity of the AFFITOPE induced antibody response

We next assessed the specificity of the Abs induced in more detail. Neither AD01-, AD02- nor Aß1-6-induced plasma samples reacted with irrelevant control peptides such as Aß11-20 offered as BSA conjugates (ELISA, not shown). The reactivity of AD01- and AD02-induced Abs towards murine Aß was limited and comparable, whereas the signal obtained with Aß1-6-induced sera was 4 times higher (Fig. 1E). Interestingly, while AD01-elicited plasma samples reacted strongly with AD01 offered as BSA conjugate in an ELISA setting they barely did so with AD02-BSA; the opposite was true for AD02-induced samples (approx. 13-fold difference, see Fig. 1F). This lacking reactivity towards the respectively other AFFITOPE while retaining reactivity towards the Aß-N-terminus is most probably explained by the fact that the amino acid sequences of the two AFFITOPEs tested in this experiment differ from each other by 67% (n = 4/6aa) but show a similar difference to the native Aß sequence of 50% (n = 3/6aa).

### Exclusion of APP reactivity

**Figure 2:**
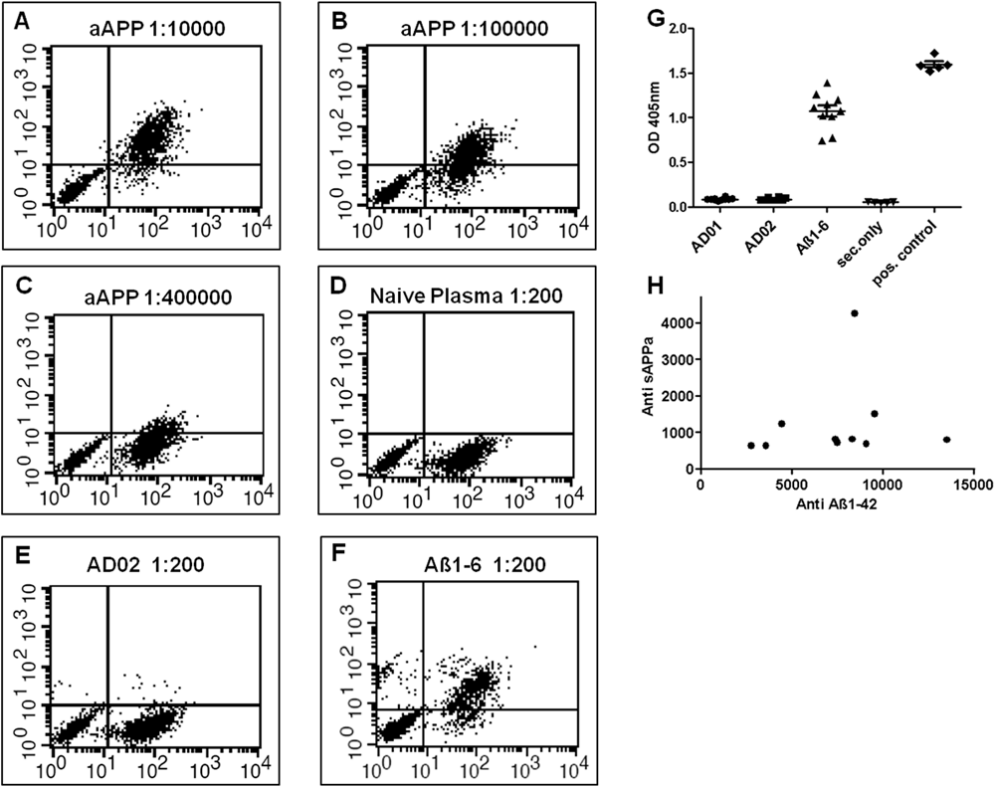
AFFITOPE induced Antibodies spare APP and sAPPa. Mice were s.c. injected 3 times at a 2-week interval with AD01-, AD02- or Aß1-6-conjugate vaccines adjuvanted with ALUM. Plasma was taken at sacrification. A-D depict controls for full length APP-specific FACS analysis using the APP-specific mAb mP2-1 (A-C) or naïve plasma (D); E and F show two exemplaric analyses of AD02 and Aß1-6 induced plasma in this assay. G and H depict analysis of immune responses against sAPPa following vaccination (n = 10 animals/treatment group) by peptide ELISA. For analysis of the presence of APP specific antibodies, the % of cells shifting in the non-APP expressing population and the % of cells shifting in the presence of the secondary antibody only were subtracted from the % of APP-positive cells shifting as indicator of APP binding. The assay threshold was set to 5%. Positive controls (A-C) show an Ab dose dependent (LOD of ≤1ng/ml mAb) shift of the APP positive but not of the APP-negative population. No reactivity was seen with plasma from naïve animals (D). AD02-induced samples show no reactivity to the APP-positive as well as APP-negative populations (E) whereas Aß1-6 induced sera show a specific shift at a dilution of 1/200 in the APP-positive cell population (upper right quadrant) with only very limited reactivity to the non-APP expressing cell population (lower and upper left quadrant; F). Neither AD01 nor AD02 was found to elicit sAPPa-specific Abs (G). On the contrary, following Aß1-6 immunization, sera of all 10 animals were shown to cross-react with sAPPa (G). A correlation analysis for anti Aß1-42 and anti sAPPa reactivity of plasma samples from animals undergoing Aß1-6 immunization (H) fails to detect a significant correlation indicating a highly individual response against sAPPa (Pearson r = 0.1534; R²= 0.02354); Titers determined were calculated based on ODmax/2 values. aAPP…anti APP specific Ab, neg.contr… is secondary Ab only; AD01, AD02 is AD01- and AD02-vaccine induced plasma, Aß1-6 is Aß1-6 vaccine induced plasma, sec.only… secondary Ab only, pos. control…. mAb P2-1

Abs used to identify the AD01/AD02 AFFITOPE-family were characterized by recognition of the Aβ-N-terminus and a lack of reactivity with full length APP, the precursor of Aβ. To check whether, as intended, AD01- and AD02-induced Abs would mirror this characteristic of the paternal mAbs, plasma of AFFITOPE-vaccinated animals were analyzed for APP-binding employing a FACS assay based on CHO-cells expressing human APP on their surface. Plasma of immunized animals were analyzed in comparison to APP-specific mAbP2-1, which showed a dose dependent signal (Fig. 2A-C). Of note, such a signal was not seen in plasma from AD01 (n = 8) or AD02 immunized animals (n = 30; representative example in Fig. 2E and data not shown). By contrast, a substantial portion of plasma from Aβ1-6-immunized animals, n = 6/30, was found to contain APP-specific Abs (Fig. 2F).

In addition, plasma samples were also tested for Abs directed against sAPPa, an important mediator of APP-function. None of the AFFITOPE samples tested (n = 20; Fig. 2G) contained sAPPa specific Abs detectable by ELISA. This differentiated them from Aß1-6-based vaccines, which induced sAPPa-reactive Abs in all animals tested (n = 10/10; Fig. 2G). Interestingly, this APP cross-reactivity was not directly correlated with the absolute anti-Aß titer in these samples (Spearman-Rank-Correlation r = 0.4316, p = 0.2129; Fig. 2H), implying that this reactivity against cleaved forms of APP is a unique feature of a fraction of Abs present within the oligoclonal response elicited by Aß1-6-based vaccines.

As a third method to assess the potential cross reactivity of AD01- and AD02-induced antibodies to human APP/sAPPa and an APP-eGFP fusion protein, a Western blot analysis was performed (see Appendix, S2A Fig.). In this assay, both AD01- and AD02 induced plasma samples failed to detect full length human APP/sAPPa in brain extracts from 12 month old Tg2576 animals or in cell extracts from CHO cells stably expressing a fusion protein of human APP and eGFP (also used in the FACS based analysis mentioned above). As expected the APP specific positive control antibody 22C11 was able to detect APP/sAPPa and APP-eGFP using this method.

### Differential reactivity towards Aß-aggregation states

**Figure 3:**
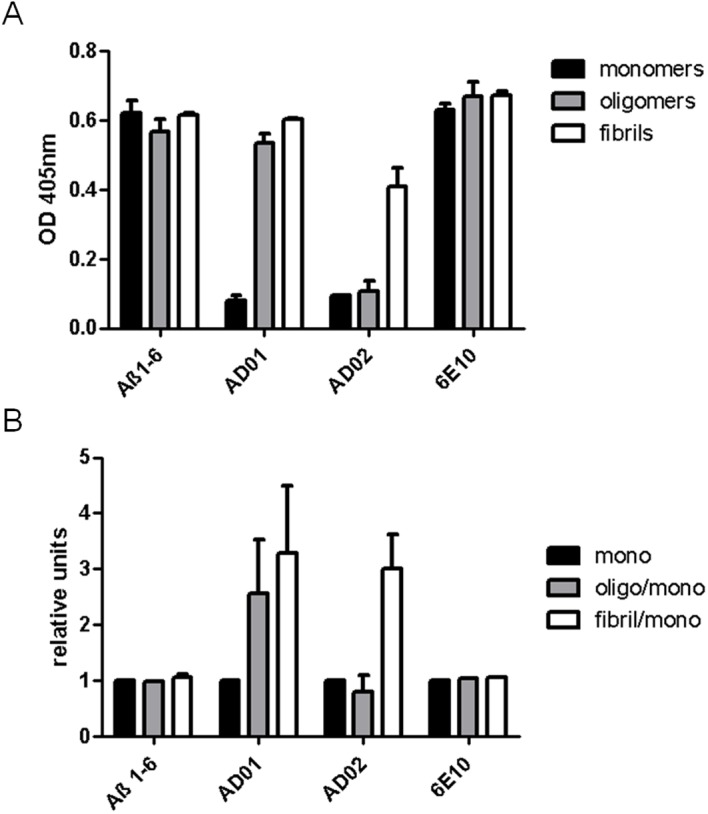
AFFITOPE induced antibodies differ in their reactivity towards aggregated forms of Aβ. A) Reactivity of Aβ1-6-, AD01- and AD02-induced Abs towards Aβ in various aggregation states. Bars represent the means of OD values (at 405nm) of individual plasma samples (duplicates) derived from single animals immunized with Aß1-6, AD01 or AD02;. B) Relative units of values from monomeric- and either oligomeric- or fibrillar Aß detection by plasma samples from Aß1-6- or AFFITOPE-treated animals are depicted (mean+/-SEM, n = 5 samples/vaccine). Levels around one indicate a similar OD whereas values above one indicate a predominant binding to either oligomers or fibrils as compared to monomeric Aß1-42. Reactivity of sera was tested against seedless monomeric, oligomeric and fibrillar Aß1-42, respectively. Purity of the preparations was comparable and exceeded 90%. The monoclonal Ab 6E10 (Signet) was used as positive control.

To characterize AD01- and AD02-induced plasma samples with regard to their reactivity towards defined Aβ-aggregation states we devised ELISA systems covering monomers, oligomers and fibrils. 6E10, known to bind Aβ in all its aggregation states (Fig 3), was used as standard. Of note, the patterns of reactivity seen with AD01, AD02 and Aβ1-6 were found to differ substantially. Aβ1-6-induced antibodies behaved like 6E10 reacting equally well with all Aβ-aggregation states tested (Fig. 3). By contrast, AFFITOPE-elicited Abs exhibited a differential recognition pattern of the various Aβ-aggregation states. AD01-induced plasma reacted with aggregated (both oligomers and fibrils) but not with monomeric Aβ. AD02-induced sera were found to recognize fibrillar Aβ only (Fig. 3) and showed only limited reactivity towards oligomeric Aß preparations and no reactivity with monomeric Aβ using ELISA based analyses.

In a second set of experiments, Aß aggregate specificity of AD01 and AD02 induced antibodies was analysed employing peptide ELISAs with aggregated Aß both as bait coated on the ELISA plate and as peptide used as competitor for antibody binding to the immobilized Aß aggregates (see Appendix, S2C Fig.). Indeed this competition experiment revealed a concentration dependent, specific reduction of the binding to aggregates and hence further substantiates the claim of selective aggregate recognition by AFFITOPE induced antibodies.

In addition, AD01 and AD02 induced antibodies were also tested for binding to monomeric, dimeric and aggregated Aß by Western blot analyses. As suggested by ELISA results (see Fig. 3), AD02 induced antibodies showed a lack of reactivity to monomeric and dimeric Aß and against low molecular weight (MW) Aß aggregates (<100kD). Reactivity could only be detected to high MW aggregates (>100kd, see Appendix, S2B Fig.). In contrast to ELISA based results, AD01 induced antibodies displayed reactivity to monomeric and dimeric Aß probably due to different sensitivity of the assays used. In line with previous results (see Fig. 3 and Appendix, S2B Fig.) they also reacted against all aggregated Aß forms present on Western blots similar to the non-confomer specific control antibody 4G8 (see Appendix; S2B Fig.).

**Figure 4:**
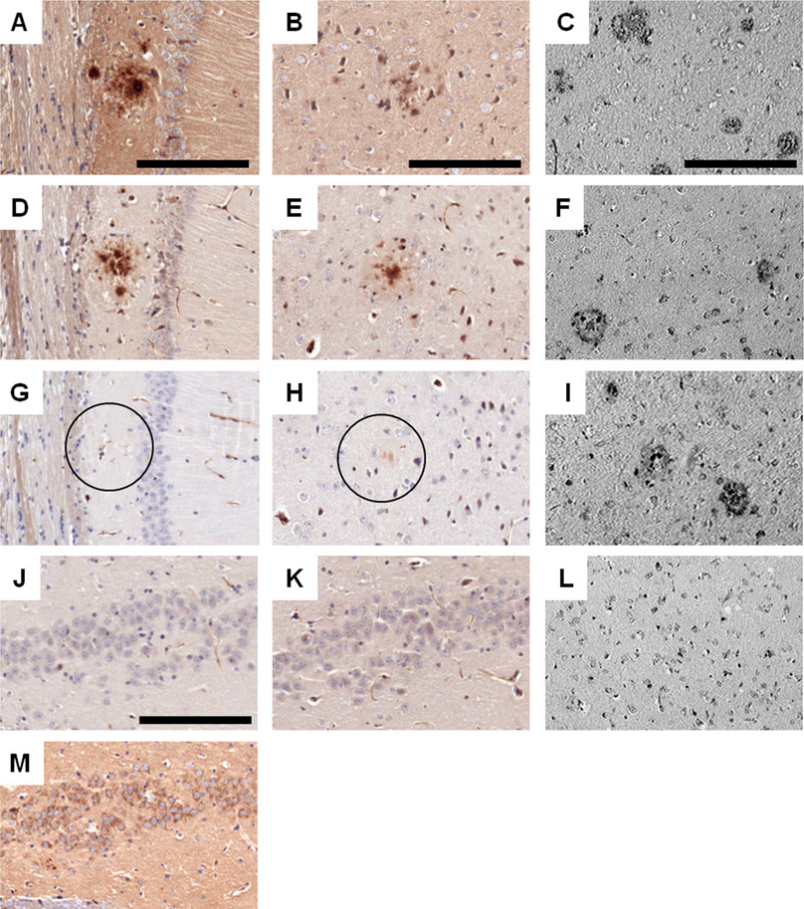
AFFITOPE-induced antibodies detect amyloid deposits but spare neuronal APP on murine and human brain sections. Sections prepared from the hippocampus (A, D, G, J, K, M) and the cortex (B, E, H), of a 12 month old Tg2576 mouse were incubated with plasma of AD01- (D, J) or AD02-treated mice (E, K) and, for control purposes, with the antibodies 6E10 (A+B) and mAb 22C11 (J, K, M), recognizing Aß and full length APP. G) and H) show a loss of immunoreactivity on Tg2576 brain sections incubated with AD01- and AD02-induced samples which were mixed with the respective AFFITOPE peptide to inhibit AFFITOPE specific staining (i.e. G: AD01-induced plasma + 10µM AD01 peptide, H: AD02-induced plasma + 10µM AD02 peptide). Sections prepared from the cortex of a female AD patient (C, F, I, L Braak stage VI,) were incubated with the Aß specific control antibody BAM10 (C), AD01- (F) and AD02-induced plasma (I) or plasma from naïve control mice as negative control (L). Binding of the Abs was detected using a standard DAB immuno-histochemistry protocol. The analysis of human sections reveals a specific amyloid deposit staining of AD01-(F) and AD02-induced Abs (I) present in murine plasma similar to staining obtained by using control antibody BAM10 (C), whereas no staining was detectable with plasma derived from a naïve, untreated animal (L). Pictures were taken at a magnification of 20x. Scale bars: 200µm; circles in G and H indicate amyloid deposits devoid of amyloid specific staining by AD01- or AD02- induced antibodies following peptide-specific competition.

Furthermore, we assessed the reactivity of AD01- and AD02-induced plasma on brain tissue of Tg2576-mice and of AD-patients (n = 4) applying a standard DAB immuno-histochemical protocol [26] and using 22C11 as comparator reacting with full length APP. AD01- or AD02-elicited plasma were found to exclusively stain amyloid deposits and to spare neuronal surfaces with a comparable staining pattern as the Aß specific control antibody 6E10 in brain sections of Tg2576-mice (Fig. 4 A, B, D, E, J and K). In addition, a loss of immunoreactivity could be detected when AD01- or AD02-elicited plasma was pre-adsorbed with the respective AFFITOPE-peptides to inhibit antibody binding to amyloid plaques, indicating specificity of the AFFITOPE-induced antibody staining observed in these animals (Fig. 4G, H). Immunohistochemical analysis of APP reactivity showed an opposite staining pattern with specific reactivity on neuronal cell walls and plaque-surrounding neuritic alterations both in the hippocampus and the cortex of Tg2576 mice (Fig. 4M and data not shown)

A comparable staining pattern was seen on human AD-brain sections. The analysis of human sections of n = 4 patients revealed a specific amyloid deposit staining of AD01-(Fig. 4F) and AD02-induced Abs (Fig. 4I) present in murine plasma similar to staining obtained by using control antibody BAM10 (Fig. 4C). No staining was detectable with plasma derived from a naïve, untreated animal (Fig. 4L). These findings corroborated the Aß-specificity described above characterized by the lack of APP cross-reactivity (Fig. 2).

### AFFITOPE vaccination does not activate Aβ-specific T-cells

**Figure 5:**
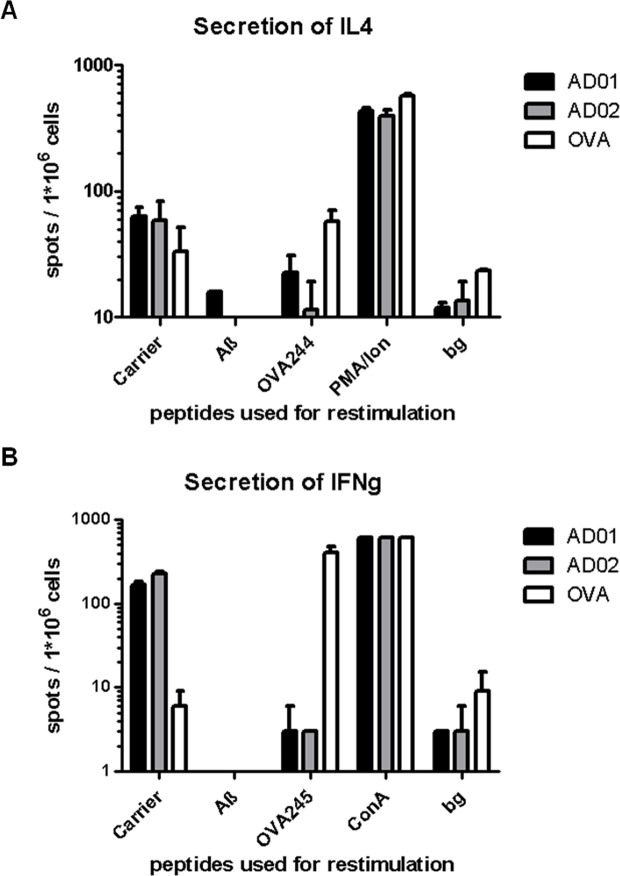
AD01 and AD02 immunization does not induce self-reactive T-Cells. Neither AD01 nor AD02 treated mice showed any sign of Aß-specific T-cell activation in two ELISPOT assays (A+B). Re-stimulation using the carrier (KLH) was resulting in a stimulation of IL4 and Interferon gamma (INFg) secretion, indicative of the presence of carrier specific T-cells following immunization with AD01 and AD02. The positive control Ovalbumin was able to induce a slightly higher Interferon gamma secretion than the carrier used in the AFFITOPE vaccines (B). A+B depict two representative ELISPOT analyses following vaccination of Ovalbumin, AD01 and AD02. A) IL4 secretion following splenocyte restimulation using carrier (KLH) and Aß compared to the controls OVA244 (TEWTSSNVMEERKIKV; MHC class II restricted to demonstrate Ovalbumin induced T-cells) and PMA/ionomycin (PMA/Ion); B) IFNg secretion following splenocyte restimulation compared to the positive controls OVA245 (SIINFEKL; MHC class I restricted to demonstrate Ovalbumin induced T-cells) and Concavalin A (ConA). Bg describes the background of secretion in non-stimulated cells in this assay. Numbers are the total number of spots per million of cells seeded on the ELISPOT plates.

**Figure 6:**
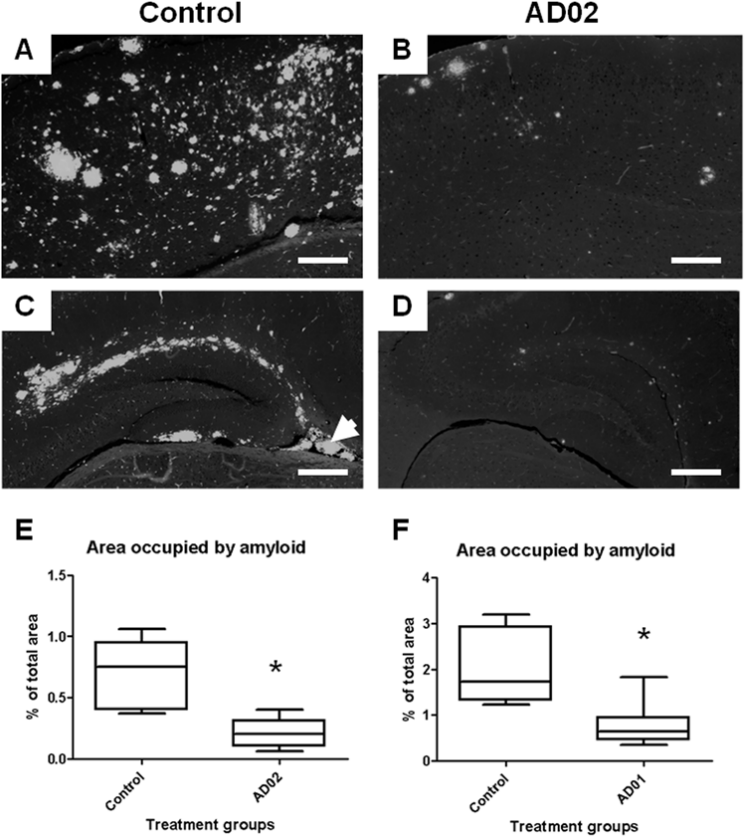
AFFITOPE immunization reduces cerebral amyloid deposition in Tg2576 mice. Groups of Tg2576 mice (n = 10/group) received 6 monthly injections of KLH/ALUM or AD01-, AD02-conjugate vaccines. Brains were isolated, 8 weeks after the 6^th^ immunization. Quantification of the relative total brain area covered by amyloid deposits (in % of total tissue analyzed) is based on immuno-fluorescence staining using the Aß specific mAb 3A5. Representative subregions of the cortex (A, B) and dentate gyrus (C, D) of controls (A, C) and AD02- (B, D) immunized mice are shown. E) AD02-vaccination reduces the relative area covered by amyloid deposits compared to controls by 70% (diffuse and dense cored amyloid; p˂0.05). F) AD01 vaccination reduces the relative area covered by amyloid deposits compared to controls by 62% (diffuse and dense cored amyloid; p˂0.05). Box plots in E and F show minimum, 25% percentile, median, 75% percentile and maximum. Asterisks in E+F indicate statistical significant difference (p<0.05); Arrowhead in C indicates unspecific fluorescence from a cerebral vessel. Scale bar: 200µM; pictures taken at 10x magnification

**Figure 7:**
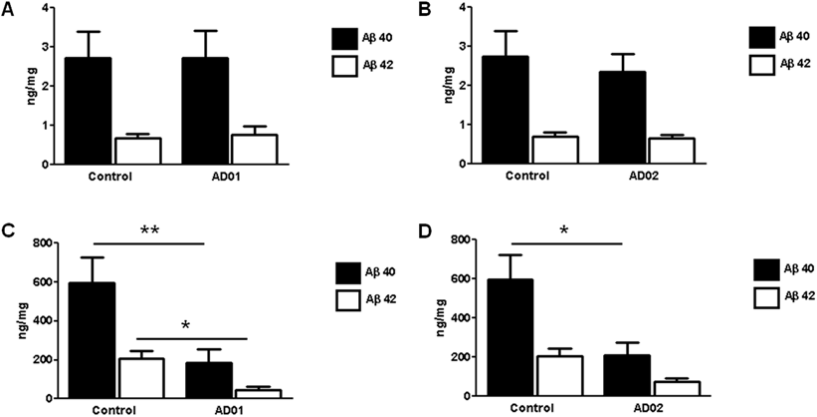
AFFITOPE immunization reduces cerebral amyloid levels in Tg2576 mice (ELISA). Groups of Tg2576 mice (n = 10/group) received 6 monthly injections of KLH/ALUM or AD01-, AD02-conjugate vaccines. Brains were isolated, 8 weeks after the 6^th^ immunization, extracted and soluble and insoluble brain fractions were subjected to Human Aß40 and Human Aß42 ELISA (EMD-Milipore, USA) analysis. Neither AD01- (A) nor AD02 treated animals (B) showed a significant change of soluble Aß1-40 and Aß1-42 following immunotherapy as compared to control immunized animals. Insoluble Aß was reduced significantly following immunotherapy. C) AD01 treated animals showed a 69% reduction of Aß1-40 levels (p = 0.005) and a 78% reduction of Aß1-42 (p = 0.015), respectively. D) For AD02 a 60% reduction of Aß1-40 (p = 0.033) and a 62% (p = 0.056) reduction of Aß1-42 could be detected. Results are expressed as average ± SEM and are given as ng/mg total protein. Black bars represent Aß1-40 and white bars represent Aß1-42 values. Asterisks in C+D indicate statistical significant difference (*…p<0.05, **…p<0.01);

AD01 and AD02 are 7 amino acids long (6 containing the mimicry information +1 residue used for conjugation). Therefore, these AFFITOPE peptides should be too short to bind to MHC molecules and activate peptide specific T-cells. In addition, their amino acid sequences differ from the one of the N-terminus of Aβ. To formally test whether conjugate vaccines containing AFFITOPEs AD01 and AD02 would activate AFFITOPE-peptide or Aβ-specific T-cells, splenocytes of immunized, non-transgenic animals were analyzed by ELISPOT. To this end, groups of n = 6 C56BL/6 mice were immunized 3 times at 2 week-intervals with AD01-KLH, AD02-KLH or ovalbumin (OVA). One week after the last immunization, splenocytes were isolated and stimulated in vitro with the carrier (KLH), Aβ or ovalbumin-derived MHC class I- (IFNg assay) and MHC class II (IL-4 assay) binding peptides. Cultures were assessed for IL-4- and IFNg-producing cells, which, given the stimulation conditions, reflect T-lymphocytes that had been primed during vaccination. Assay controls included stimulation with PMA/ionomycin (IL-4 assay) and concanavalin A (IFNg assay) and confirmed cell viability/functionality (Fig. 5A and B). Restimulation with the carrier protein demonstrated that both AFFITOPE vaccines had led to the induction of a KLH-specific T-cell response. Such a response was not evident in OVA-immunized animals. However, in vitro stimulation of splenocytes derived from AD01 and AD02-immunized animals with either AD01- or AD02-peptides as well as with recombinant Aβ did not yield a signal over background confirming the expected inability of the two AFFITOPEs of activating either AFFITOPE peptide- as well as Aβ-specific T-cells. This view is supported by experimental evidence from transgenic animals undergoing active immunotherapy using AD01 and AD02: To test whether AD01 and AD02 immunotherapy would lead to brain infiltration of T-cells, brain sections of n = 20 AD02-immunized-, n = 9 AD01-immunized-, and n = 10 carrier-treated Tg2576 mice were subjected to immunohistochemical examination using CD3 specific antibodies to detect potential CD3+ T cells (see Appendix, S3 Fig.). Despite that fact that in most of the AD01- and AD02- treated animals immunisation had resulted in a reduction of amyloid deposition (see Fig. 6 and 7), none of the brains was found to be infiltrated by CD3+ T cells.

### Both vaccine candidates lower cerebral Aß without triggering cerebral amyloid angiopathy (CAA) or micro-hemorrhages (MH)

To test whether AD01 and AD02 would lower cerebral amyloid load, groups of 6-months old Tg2576-mice (n = 10/group) were vaccinated 6x at monthly intervals with either vaccine (independent experiments), and sacrificed at 14 months of age. Their brains were assessed for diffuse and dense-cored plaques by IF-staining using monoclonal antibody 3A5. Cortical as well as hippocampal sections of KLH/ALUM-injected controls were covered by numerous amyloid plaques. They covered on average 2.00% (AD01 experiment) and 0.69% (AD02 experiment) of the area analyzed. By contrast, respective brain areas of AD01- and AD02-immunized Tg2576-mice contained significantly less deposits (Fig. 6 A-D) covering 0.21% (Fig. 6E) and 0.77% (Fig. 6F), respectively. Thus, AD01 reduced the area covered by amyloid by 62% (p<0.05) and AD02 by 70% (p<0.05).

In addition to the analysis of amyloid deposition in situ we also assessed the effect of AFFITOPE vaccination on the cerebral level of Aß1-40 and Aß1-42 by peptide ELISA. Therefore, brain samples of AD01 and AD02 treated Tg2576 animals were extracted and soluble and insoluble brain fractions were subjected to Human Aß40 and Human Aß42 ELISA (EMD-Milipore, USA) analysis. Neither AD01 nor AD02 treated animals showed a significant change of soluble Aß1-40 and Aß1-42 following immunotherapy (see Fig. 7A and B). In contrast for both vaccines, insoluble Aß was reduced significantly following immunotherapy (see Fig. 7C and D). For AD02 a 60% reduction of Aß1-40 (p<0.05) and a 62% (p = 0.056) reduction of Aß1-42 could be detected. AD01 showed a reduction of 69% (Aß1-40, p<0.05) and 78% (Aß1-42, p<0.01), respectively. This differences are most probably reflecting a selective removal of aggregated and deposited Aß while soluble forms were only reduced to a low amount.

**Figure 8:**
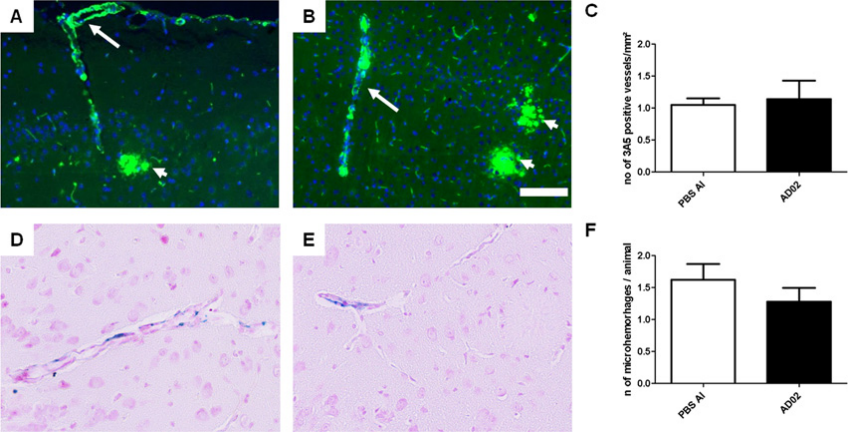
Cerebral amyloid angiopathy and microhemorrhaging are unchanged following AFFITOPE-immunization. The analysis of the incidence of amyloid bearing vessel in AFFITOPE- and control treated animals by assessing 3A5 staining in cerebral vessels reveals no significant differences (A-C). A) Example from a cortical section of a control animal. B) Example from a cortical section of an AD02-treated animal. C) Quantitative analysis demonstrating the average number of 3A5 positive vessels/mm² (avg. +/- SEM) in control (n = 9), and AD02-treated animals (n = 8). The analysis of the incidence of cerebral micro-hemorrhages in these animals by assessing Hemosiderin staining (= Prussian Blue) did not show significant differences at 14 months of age (D-F). D) Example from a cortical section of a control animal. E) Example from a cortical section of an AD02-treated animal. F) quantitative analysis demonstrating the average number of Hemosiderin positive vessels/animal in control (n = 9), and AD02 treated animals (n = 8, respectively); Arrows indicate 3A5 positive vessels, arrowheads indicate amyloid deposits. Scale bar: 50µm; pictures taken at 20x magnification

As amyloid removal appears to partially occur via the vasculature and peripheral sink mechanisms [31, 32, 33] and can be associated with enhanced micro-bleedings following active and passive immunotherapy [34], blood-vessels of relevant brain regions (cortex and hippocampus) were analyzed for their amyloid content by 3A5 staining and the same regions were assessed for microbleedings by Prussian Blue staining, respectively. At the time point assessed, the number of 3A5-positive vessels per mm^2^ is comparable for control- and AFFITOPE-treated animals (see Fig. 8) compatible with the view that AFFITOPE vaccines do not enhance CAA after repeated immunization. Moreover, the MH-number was low and comparable for both immunized and control mice (Fig. 8) indicating no effect on the occurrence of micro-hemorrhages following AFFITOPE immunization.

### Both vaccine candidates improve functional deficits of APP-transgenic mice

To evaluate the effect of AFFITOPE-vaccination on cognitive functions, we applied, MWM (AD01 and AD02) and CFC (AD02 only) analyzing spatial and contextual memory and learning in Tg2576-mice.

**Figure 9:**
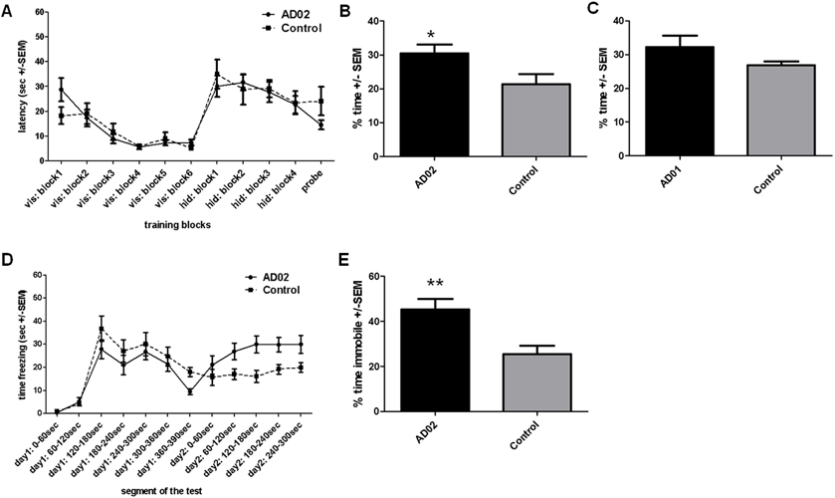
AFFITOPE vaccination improves cognitive function of Tg2576-mice. Spatial memory and learning were assessed with MWM (A-C), contextual learning and memory with CFC (D-E). Cognitive testing was initiated 4 weeks prior to sacrification in groups of AFFITOPE-immunized or Tg2576 control mice. A) Learning curves as assessed by escape latency for visible as well as hidden platform training. Both groups exhibited comparable learning capacities. B) Spatial memory assessed by probe trial using % of time spent in target quadrant at the end of MWM. AD02 vaccination improves spatial memory by 40% (p˂0.05). Similar results were obtained for distance travelled (not shown). C) Spatial memory assessed by probe trial using time spent in target quadrant at the end of MWM. AD01 vaccination increases the time spent in the target quadrant by 25% (n = 5 animals/group; p = 0.1). D) Development of the time freezing on day 1 and day 2 of the CFC-analysis revealed that AD02-treated animals show slightly lower freezing levels in response to foot shocks on day 1 (p > 0,05), but froze significantly longer during the retention phase of the CFC test at day 2 (p<0,01). E) % of time freezing at the end of CFC testing. Parameter depicted in D and E is the % of time the animals are 99% immobile during a representative 2-minute period on day two of the CFC testing paradigm. *..p<0.05; **..p<0.01.

In the MWM learning phase, both AD02- and control-treated mice (receiving KLH/ALUM) showed a similar learning capability (Fig. 9A). During probe-trials for assessing memory retention, AD02-treated mice performed significantly better than control mice (Fig. 9B, p<0.05). No differences in swim speed between the two groups were detectable during the probe trial (data not shown). Analysing the percentage of mice per group searching in the target quadrant for >25% of the time, showed that 82% of the AD02-treated animals were able to correctly remember the former platform position compared to 40% in the control group (data not shown). A similar MWM-analysis using AD01-treated animals revealed a similar improvement in spatial memory. However, while AD01-immunized animals performed better than controls, the effect did not reach statistical significance (Fig. 9C, p = 0.1).

CFC demonstrated that AD02-treated mice were superior to control animals (Fig. 9D+E). Although showing slightly lower freezing levels in response to day 1 foot-shocks (Fig. 9D), animals froze significantly more on day 2 during the representative 2 min period of the retention phase of the test (p<0.01, Fig. 9D+E). The significant improvement of AD02-treated animals was also detectable by averaging the performance in CFC during the whole 5 minutes on day 2 (p<0.05, data not shown). Taken together, these findings demonstrate AFFITOPE-vaccination to effectively reduce memory defects in Tg2576-mice in two learning paradigms.

## Discussion

The work presented aimed at generating novel Aß-targeting AD-vaccines with specific features. Specifically, they were designed to (i) trigger Abs specific for the Aß-N-terminus (ii) being selective for aggregated Aß and (iii) preclude the activation of AFFITOPE peptide- or Aß-specific T-cells. This was accomplished via the mechanism of molecular mimicry and by applying sequential selection filters. It led to the identification of two candidates, AD01 and AD02, which fulfill the predefined criteria and exhibit disease-modifying activity in the models tested.

Molecular mimicry, in terms of humoral immunity, denotes the phenomenon of Abs not only recognizing a single epitope but more than one resembling each other and, thus, being indistinguishable for the Ab. This is not uncommon. Most examples we are aware of relate to negative effects. They include autoimmune reactions as a result of bacterial- or viral infections, neoplasias (e.g., paraneoplastic CNS disorders) or vaccination (e.g., AN1792-triggered cases of meningoencephalitis) [12, 35, 36]. We explored the possibility of exploiting molecular mimicry for the development of AD-vaccines with optimized safety- and efficacy features. To this end, Abs known to bind the Aß-N-terminus (DAEFRH), were exposed to a pool of 10^9^ 6- or 7-mer peptides. In addition to DAEFRH, a total of 68 peptides were found to bind the Abs employed (frequency of cross-reactive peptides: 4.0x10^-8^). 20.5% of the hits differed at every aa-position from the original epitope, the remaining had 2 or more aa-exchanges. For all 68 peptides, binding could be competed with DAEFRH arguing for their interaction with the antigen binding sites of the Abs. Out of the 68 peptides, 17 were picked and tested for their ability to elicit Abs when administered as peptide-KLH conjugates adjuvanted with aluminum. All 17 elicited an Ab-response to the immunizing peptide, 14 of them induced Abs reacting with Aß1-10-BSA conjugates, which resemble to some extent Aß-aggregates given the high density of binding sites on BSA following conjugation. These data confirm and quantify the phenomenon of molecular mimicry for mAbs primarily known to bind to the Aß-N-terminus. They also demonstrate, at least for mice, that it is possible to reverse and hence exploit the process. That is, cross-reacting peptides, foreign to the human proteome, can trigger Abs that recognize the „original“epitope and have imprinted „additional“features, e.g. selective Aß-aggregate recognition and selective recognition of peptides derived from the same screen.

Beyond pathophysiology, design of AD-vaccines has to consider physiological functions and the dynamics of the ensuing Ab-response. While AD-pathophysiology is complex, it appears that toxicity resides within the aggregated Aß fraction affecting neurons and synapses [21, 22, 23, 24]. By contrast, monomeric Aß as well as sAPPa and APP possess physiological functions. Monomeric Aß regulates the proliferation of neural progenitors and contributes to synaptic function [17, 18, 19, 20, 37]. APP and sAPPa are involved in the development and plasticity of the nervous system, regulation of neurite outgrowth, neuronal proliferation and contribute to cognitive performance and memory [38, 39, 40, 41, 42, 43, 44, 45, 46, 47, 48, 49, 50]. Abs recognizing physiological elements of the Aß-pathway, such as the ones induced by Aß1-6-KLH, could have negative effects via various, mutually non-exclusive mechanisms: interference with the above molecules and their functions, Ab-triggered cytotoxicity. Moreover, APP/sAPPa and serum Aß would sequester such Abs thereby lowering their levels. Of note, the advantages of Abs with the above specificity may not be discernible in AD-models used, which are characterized by a strong over-expression of Aß and sAPPa. So far, AFFITOPE-vaccines are the first second-generation vaccines which report to spare binding to the above molecules. Other examples either do not provide any analyses on cross-reactivity [14, 15, 16] or report a lack of APP/sAPP reduction without providing data or an analysis of antibody binding other than on fixed tissue, therefore probably underestimating potential cross-reactivity in vivo [13].

Beyond differential targeting of Aß-variants, AD01 and AD02 reduced cerebral amyloid load by 62- and 70%, respectively. This compares favorably to conventional vaccines [13, 14, 15, 16, 51]. In addition to the IF analysis, assessing the amount of insoluble Aß showed a selective and significant reduction following AD01 and AD02 immunotherapy, whereas soluble forms of Aß1-40 and Aß1-42 were not significantly changed. This selective removal of insoluble and deposited Aß could further support the selectivity of AFFITOPE-vaccines for Aß-aggregates.

Amyloid reduction was not associated with an increase in detectable MH or CAA, as seen with other vaccines [52, 53], but with improvement of cognitive dysfunction as assessed by MWM and CFC. Furthermore, we could also demonstrate the inability of the two AFFITOPE vaccines of activating either AFFITOPE peptide- as well as Aβ-specific T-cells. This is in line with similar experimental results obtained using AFFITOPE vaccines targeting alpha Synuclein (aSyn) in animal models of synucleinopathies [54]. In these experiments, no aSyn AFFITOPE peptide- or target specific T-cells (i.e. alpha Synuclein) could be detected by ELISPOT- or immunohistochemical analyses following active immunotherapy in mice using peptide conjugate vaccines [54].

In conclusion, data presented support the feasibility of the proposed technology based on molecular mimicry. Vaccine candidates identified, AFFITOPEs AD01 and AD02, exhibit high specificity (Aß-aggregates but no monomers) defining their high safety (e.g., sparing of APP/sAPPa-recognition) and efficacy profiles. Given their disease-modifying potential both have been introduced to clinical testing in mild to moderate AD with AD02 being currently assessed in a multicentre phase II study in early AD-patients.

## Supporting Information

S1 Fig Aggregation analysis of Aβ-monomers,-oligomers and—fibrils. To assess aggregation status of Aβ-monomers,-oligomers and—fibrils, ThT Fluorescence analsyis (A) as well as Dot blot (B) and Western blot (C) were performed. (A) Monomer preparations show relative fluorescence units (RFU) close to background indicating the absence of fibrillar Aß. Oligomeric and fibrillar preparations contained ThT positivive aggregates with fibrillar preparations containing significantly more positive aggregates (RFUs >5000) than oligomeric preparations (RFUs of ca. ≤2000). (B) Dot Blot analysis using NAB 228 showed equal signals for Aβ-monomers,-oligomers and—fibrils whereas analysis using A11 did show only oligomer specific signals and failed to detect Aβ-monomer and—fibril preparations indicating that only the oligomer preparations were also containing oligomeric species, not detectable in the other two preparations. (C) Western Blot analysis using NAB 228 showed equal signals for Aβ-monomers and—oligomers. Oligomeric preparations contained Aβ‐dimers, ‐trimers and ‐tetramers as well as oligomers with a size of approx. 35–40kd (weak signal in C) in this analysis. No fibril specific signals could be detected.
(TIF)

S2 Fig Reactivity of AD01- and AD02-induced antibodies to Aß and sAPPa. The reactivity of AD01- and AD02-induced Abs towards full length APP/sAPPa/APP-eGFP as well as different forms of Aβ was assessed by Western blot analysis (A+B). Specificity of AFFITOPE-induced antibodies for aggregated Aß was assessed by competition ELISA (C). A) Western blot analysis using brain extracts form a 12 month old female Tg2576 mouse and from CHO cells stably expressing a human APP-eGFP fusion protein showed a lack of reactivity of AD01- and AD02- induced antibodies against full length APP/sAPPa and APP-eGFP fusion protein whereas the positive control antibody 22C11 (APP-specific) was able to detect APP/sAPPa and APP-eGFP, respectively. B) Western Blot analysis of aggregated recombinant Aß revealed a lack of reactivity of AD02-induced Abs against mononmeric and dimeric Aß as well as low molecular weight (MW) Aß aggregates (<100kD). AD02-induced Abs react predominantly against high MW Aß aggregates (>100kD). AD01 induced antibodies, as the non-confomer specific antibody 4G8 showed binding to Aβ-monomers,—dimers, as well as low and MW Aß aggregates. C) ELISA experiment demonstrating the selectivity of AD01- and AD02- induced antibodies for aggregated Aß by concentration dependent, specific competition using aggregated Aß. Bars represent the means of OD values (at 405nm) of individual samples derived from single animals immunized with AD01 or AD02. Reactivity of sera was tested against aggregated Aß1-42 immobilised on ELISA plates (1µM). Competition was done using plasma samples (dilution of 1/100) and aggregate concentrations of 0.5µg/ml and 1µg/ml, respectively. A: 1…Tg2576 brain extract; 2…CHO APP-eGFP cell extract; B: m+d…Aß monomer and dimer, l+h…low and high MW Aß aggregates; C: sec. only… secondary antibody used as background control for the ELISA; grey and black bars indicate OD values for AD01 (grey) and AD02 (black) induced antibodies (+/- aggregated Aß)
(TIF)

S3 Fig T-cell response to immunization with AD01 and AD02. Immunostaining of T-cells present in the perivascular space with an anti-CD3 antibody. No CD3-positive cells were observed in brains of Control (A), AD01 (B) or AD02 (C) immunized animals. CD3 positive cells could be detected in murine splenic tissue sections used as positive controls for staining (D). Pictures in A-C show CA1 region of brains from 14 month old Tg2576 animals undergoing immunotherapy. Per mouse, a total of ≤20 individual brain sections were assessed. Scale bar = 50 μm, pictures taken at a 20x magnification.
(TIF)

S1 Appendix Materials And Methods. (DOCX)
