# Supplementary figures and images for: Tailoring the Antibody Response to Aggregated Aß Using Novel Alzheimer-Vaccines
Source: PLoS One. 2015 Jan 22;10(1):e0115237. doi: 10.1371/journal.pone.0115237 (PMC4303436; doi:10.1371/journal.pone.0115237)

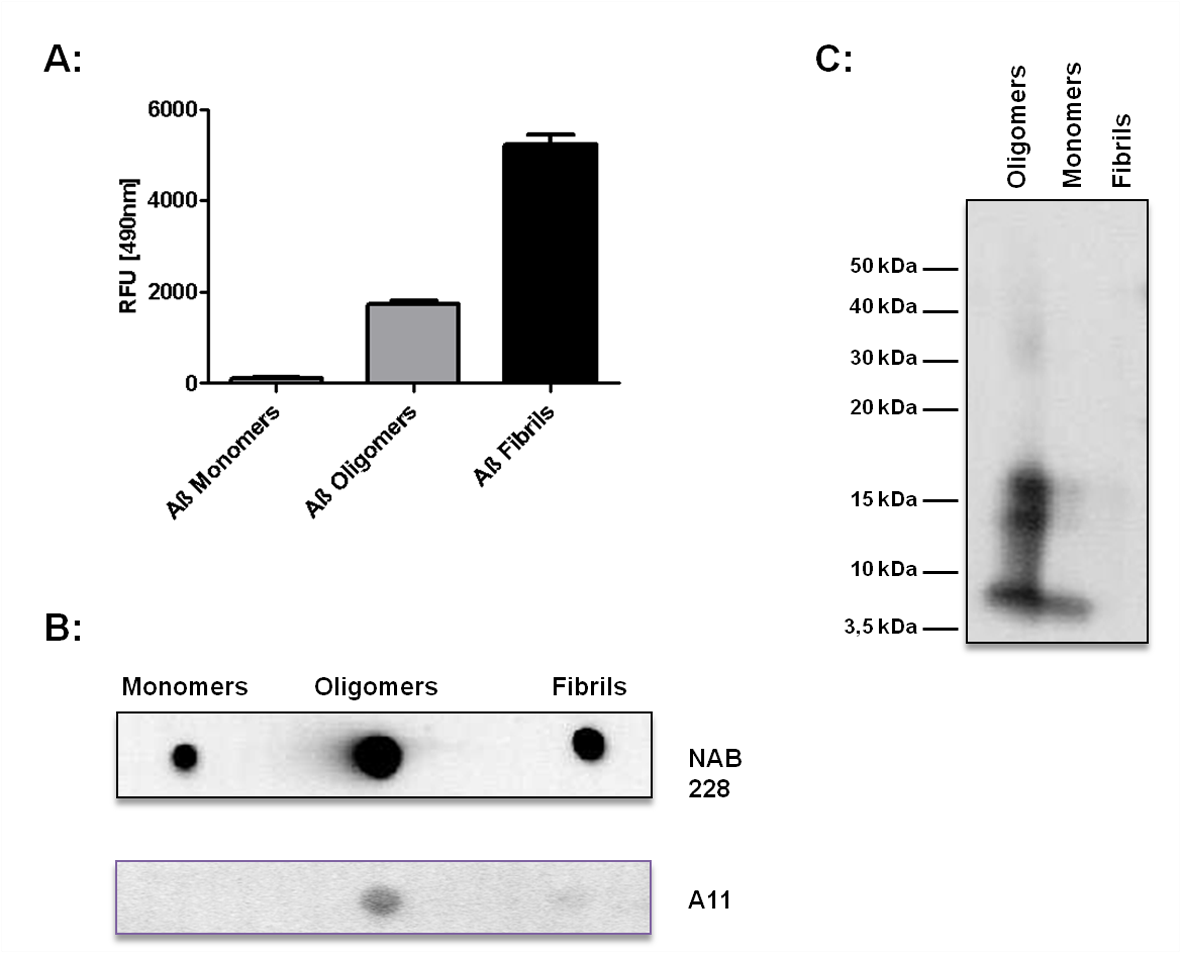

Supplement: S1 Fig — To assess aggregation status of Aβ-monomers,-oligomers and—fibrils, ThT Fluorescence analsyis (A) as well as Dot blot (B) and Western blot (C) were performed. (A) Monomer preparations show relative fluorescence units (RFU) close to background indicating the absence of fibrillar Aß. Oligomeric and fibrillar preparations contained ThT positivive aggregates with fibrillar preparations containing significantly more positive aggregates (RFUs >5000) than oligomeric preparations (RFUs of ca. ≤2000). (B) Dot Blot analysis using NAB 228 showed equal signals for Aβ-monomers,-oligomers and—fibrils whereas analysis using A11 did show only oligomer specific signals and failed to detect Aβ-monomer and—fibril preparations indicating that only the oligomer preparations were also containing oligomeric species, not detectable in the other two preparations. (C) Western Blot analysis using NAB 228 showed equal signals for Aβ-monomers and—oligomers. Oligomeric preparations contained Aβ‐dimers, ‐trimers and ‐tetramers as well as oligomers with a size of approx. 35–40kd (weak signal in C) in this analysis. No fibril specific signals could be detected. (TIF) [file pone.0115237.s001.tif]

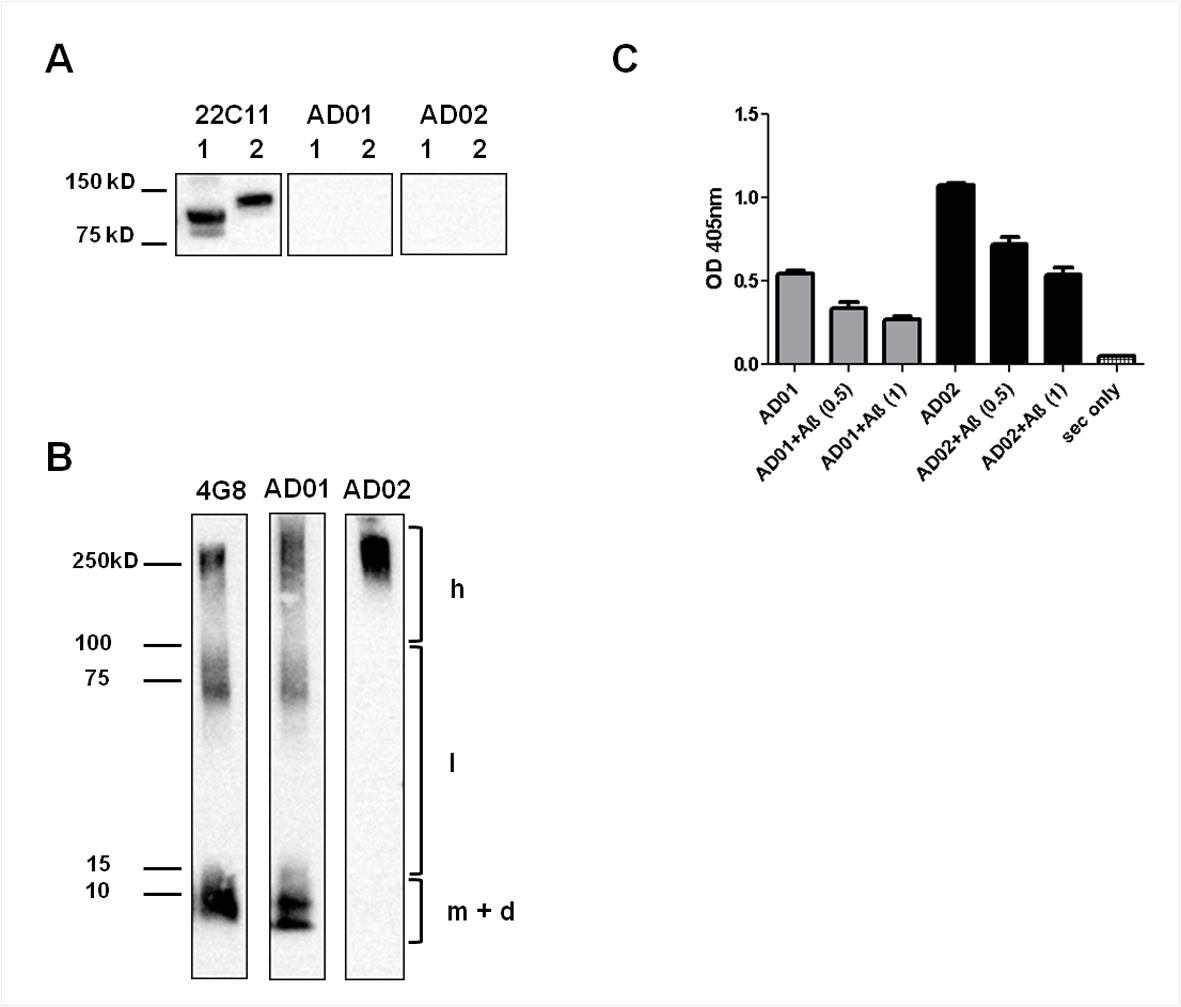

Supplement: S2 Fig — The reactivity of AD01- and AD02-induced Abs towards full length APP/sAPPa/APP-eGFP as well as different forms of Aβ was assessed by Western blot analysis (A+B). Specificity of AFFITOPE-induced antibodies for aggregated Aß was assessed by competition ELISA (C). A) Western blot analysis using brain extracts form a 12 month old female Tg2576 mouse and from CHO cells stably expressing a human APP-eGFP fusion protein showed a lack of reactivity of AD01- and AD02- induced antibodies against full length APP/sAPPa and APP-eGFP fusion protein whereas the positive control antibody 22C11 (APP-specific) was able to detect APP/sAPPa and APP-eGFP, respectively. B) Western Blot analysis of aggregated recombinant Aß revealed a lack of reactivity of AD02-induced Abs against mononmeric and dimeric Aß as well as low molecular weight (MW) Aß aggregates (<100kD). AD02-induced Abs react predominantly against high MW Aß aggregates (>100kD). AD01 induced antibodies, as the non-confomer specific antibody 4G8 showed binding to Aβ-monomers,—dimers, as well as low and MW Aß aggregates. C) ELISA experiment demonstrating the selectivity of AD01- and AD02- induced antibodies for aggregated Aß by concentration dependent, specific competition using aggregated Aß. Bars represent the means of OD values (at 405nm) of individual samples derived from single animals immunized with AD01 or AD02. Reactivity of sera was tested against aggregated Aß1-42 immobilised on ELISA plates (1µM). Competition was done using plasma samples (dilution of 1/100) and aggregate concentrations of 0.5µg/ml and 1µg/ml, respectively. A: 1…Tg2576 brain extract; 2…CHO APP-eGFP cell extract; B: m+d…Aß monomer and dimer, l+h…low and high MW Aß aggregates; C: sec. only… secondary antibody used as background control for the ELISA; grey and black bars indicate OD values for AD01 (grey) and AD02 (black) induced antibodies (+/- aggregated Aß) (TIF) [file pone.0115237.s002.tif]

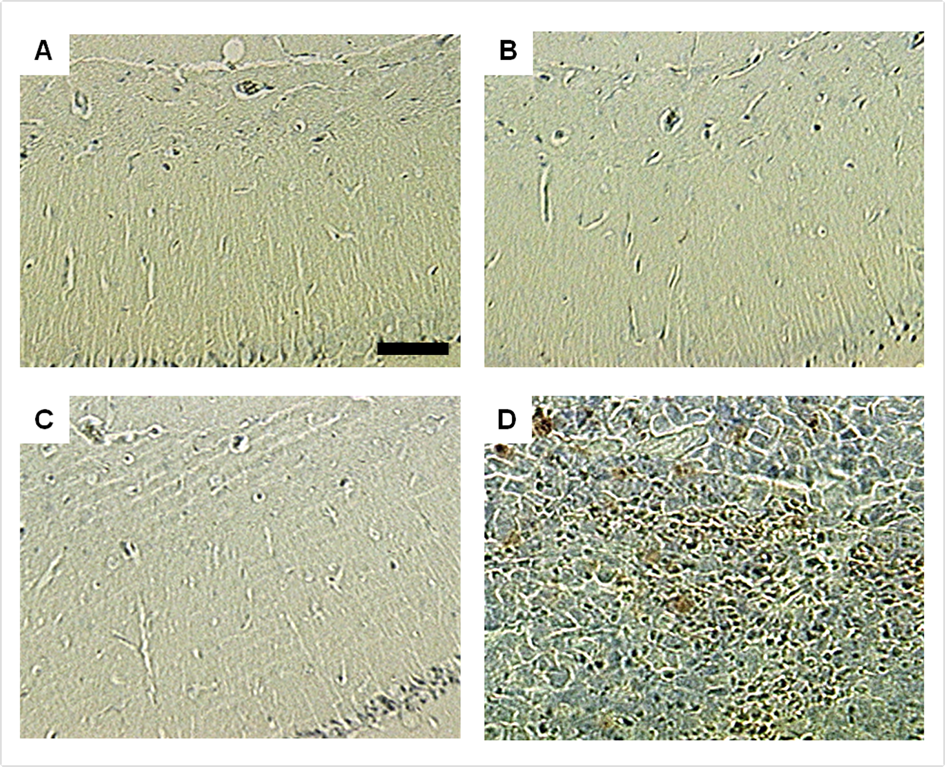

Supplement: S3 Fig — Immunostaining of T-cells present in the perivascular space with an anti-CD3 antibody. No CD3-positive cells were observed in brains of Control (A), AD01 (B) or AD02 (C) immunized animals. CD3 positive cells could be detected in murine splenic tissue sections used as positive controls for staining (D). Pictures in A-C show CA1 region of brains from 14 month old Tg2576 animals undergoing immunotherapy. Per mouse, a total of ≤20 individual brain sections were assessed. Scale bar = 50 μm, pictures taken at a 20x magnification. (TIF) [file pone.0115237.s003.tif]
