# Supplementary material for: Tailoring the Antibody Response to Aggregated Aß Using Novel Alzheimer-Vaccines
Source: PLoS One. 2015 Jan 22;10(1):e0115237. doi: 10.1371/journal.pone.0115237 (PMC4303436; doi:10.1371/journal.pone.0115237)
Supplement: S1 Appendix — (DOCX) [file pone.0115237.s004.docx]

# **Appendix:** Materials And Methods

**Determination of Aß Monomers, oligomers and fibrils**

In order to analyse the aggregation status of the Aß Monomer, oligomer and fibril preparations ThT Fluorescence analsyis as well as Dot blot and Western blot analysis were done.

For determination of the relative fibril content, a Thioflavin T (ThT; Sigma‐Aldrich, USA) fluorescence assay was used. Monomer‐, Oligomer‐ and Fibril preparations (4μM each) were incubated with 10μM ThT on 384‐Well Polypropylenplates (Greiner Bio‐One, Austria) and the relative fluorescence intensity (relative fluorescence units, RFU; λex = 490 nm; λem = 440 nm) was measured. Determination of RFUs was based on mean values of four independent measurements and background values for ThT fluorescence and buffer were subtracted.

For dot blot analyses, Aβ-monomers, -oligomers and –fibrils were spotted on nitrocellulose membranes (GE Healthcare, UK). Western Blot analysis of Aβ-monomers, -oligomers and –fibrils was performed using semi-native 4-12% Bis-Tris SDS-PAGE gel electrophoresis (Invitrogen, USA) and blotting onto polyvinylidene fluoride (PVDF) membranes. Detection was performed using the non-confomer specific mAb NAB228 and the oligomer specific antibody A11 (Life Technologies) and the SuperSignal West Dura kit (Thermo Scientific, USA).

**Determination of reactivity against Aß and APP/sAPPa/APP-eGFP**

The reactivity of AD01- and AD02-induced Abs towards monomeric, dimeric, oligomeric/aggregated Aβ as well as human full length APP/sAPPa and a full length human APP-eGFP fusion protein was assessed by Western blot analysis (please note: APP-eGFP has a higher MW than full length APP due to the ca.30kD eGFP present in the fusion protein). Specificity of AFFITOPE-induced antibodies for aggregated Aß was additionally assessed by Western blot and competition ELISA. Tg2576 brain- (soluble fraction) and cell extracts of CHO cells stably expressing APP-eGFP were prepared as described in the material and methods sections. Western blot analyses were performed as described above with the exception of ECL-Plus which was used as color substrate. Control antibodies used were the specific monoclonal antibodies 4G8 (Covance, USA, Aß) and 22C11 (APP, Merck, USA). Recombinant aggregated Aß was used at a concentration of 1µg/µl.

Competition ELISA was performed as described in the material and methods sections with the exception that aggregated Aß was used for coating and competition. Aggregated Aß concentrations used for coating were 1µM and for competition 0.5µg/ml and 1µg/ml Aß was added to the plasma samples. Plasma samples for competition ELISA were used at a dilution of 1/100.

**Determination of T cell responses: anti CD3 immuno-histochemistry**

Tg2576 mice were immunized s.c. 6 times in monthly and sacrificed at 14 months of age, brains were collected and hemispheres separated. One hemisphere was fixed in 4% Paraformaldehyde (PFA,Sigma Aldrich, USA), dehydrated and paraffin-embedded. Brain tissue was sectioned at 7μM using a sliding microtome (Leitz, Germany) and sections were mounted on Superfrost Plus Slides (Menzel, Germany). For CD3-specific staining, brain sections of immunized Tg2576 were processed for analysis using hamster mAb CD3 epsilon antibody 145-2C11 (Abcam). All secondary reagents used were obtained from Vector Labs (USA). For CD3 analysis, color reactions were performed using DAB-substrate Kit.
